# 1*H*-1,2,4-Triazole-3-carboxamide

**DOI:** 10.1107/S1600536808014013

**Published:** 2008-05-17

**Authors:** Qing-Ruo Xie

**Affiliations:** a Department of Biological and Chemical Engineering, Guangxi University of Technology, Liuzhou 545006, People’s Republic of China

## Abstract

Planar mol­ecules of the title compound, C_3_H_4_N_4_O, are organized into sheets by extensive N—H⋯O and N—H⋯N hydrogen bonding in the (101) plane of the crystal structure. These hydrogen bonds may also stabilize the mol­ecule in the *Z* form. The title compound is in the amide form, as shown by the C=O bond length [1.252 (2) Å].

## Related literature

Azo compounds are widely utilized as dyes and analytical reagents (Malinauskas *et al.*, 2000 ▶). The inter­actions of amide groups are of inter­est because of their importance in biochemical systems (Crespo *et al.*, 2005 ▶).
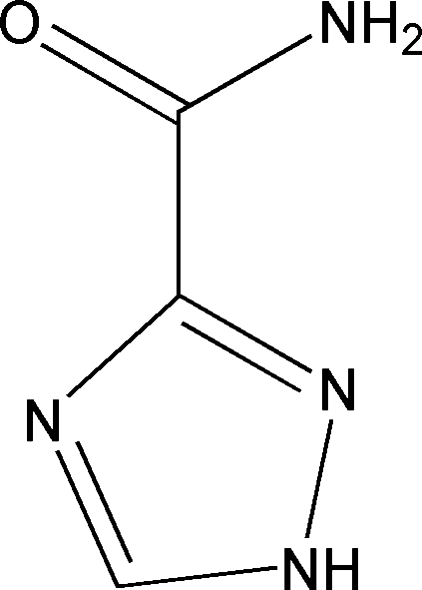


## Experimental

### 

#### Crystal data

C_3_H_4_N_4_O
*M* *_r_* = 112.10
Monoclinic, 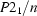

*a* = 3.6944 (4) Å
*b* = 17.527 (3) Å
*c* = 7.0520 (17) Å
β = 94.4670 (10)°
*V* = 455.24 (14) Å^3^
*Z* = 4
Mo *K*α radiation
μ = 0.13 mm^−1^
*T* = 298 (2) K
0.22 × 0.18 × 0.09 mm

#### Data collection

Bruker SMART CCD diffractometer
Absorption correction: multi-scan (*SADABS*; Sheldrick, 1996 ▶) *T*_min_ = 0.972, *T*_max_ = 0.988
2199 measured reflections
807 independent reflections
657 reflections with *I* > 2σ(*I*)
*R*_int_ = 0.020

#### Refinement

*R*[*F* ^2^ > 2σ(*F* ^2^)] = 0.035
*wR*(*F* ^2^) = 0.101
*S* = 1.05
807 reflections
73 parameters
H-atom parameters constrained
Δρ_max_ = 0.13 e Å^−3^
Δρ_min_ = −0.23 e Å^−3^

### 

Data collection: *SMART* (Bruker, 2003 ▶); cell refinement: *SAINT* (Bruker, 2003 ▶); data reduction: *SAINT*; program(s) used to solve structure: *SHELXS97* (Sheldrick, 2008 ▶); program(s) used to refine structure: *SHELXL97* (Sheldrick, 2008 ▶); molecular graphics: *SHELXTL* (Sheldrick, 2008 ▶); software used to prepare material for publication: *SHELXTL*.

## Supplementary Material

Crystal structure: contains datablocks I, global. DOI: 10.1107/S1600536808014013/cs2075sup1.cif

Structure factors: contains datablocks I. DOI: 10.1107/S1600536808014013/cs2075Isup2.hkl

Additional supplementary materials:  crystallographic information; 3D view; checkCIF report

## Figures and Tables

**Table 1:** Hydrogen-bond geometry (Å, °)

*D*—H⋯*A*	*D*—H	H⋯*A*	*D*⋯*A*	*D*—H⋯*A*
N1—H1*A*⋯O1^i^	0.86	2.21	3.065 (2)	173
N1—H1*B*⋯N4^ii^	0.86	2.22	3.010 (2)	154
N2—H2⋯O1^iii^	0.86	2.07	2.909 (2)	163
N2—H2⋯N3^iii^	0.86	2.54	3.055 (2)	120

Symmetry codes: (i) 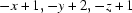; (ii) 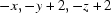; (iii) 
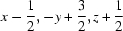
.

**Fig. 1.**
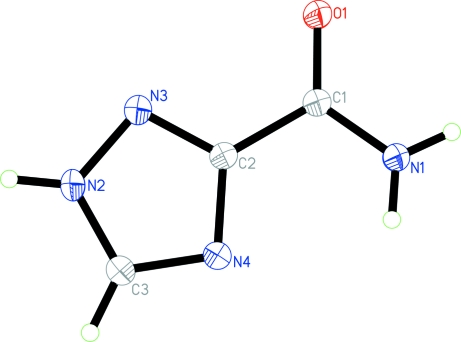
The structure of the title compound showing 30% probability displacement ellipsoids and the atom-numbering scheme.

**Fig. 2.**
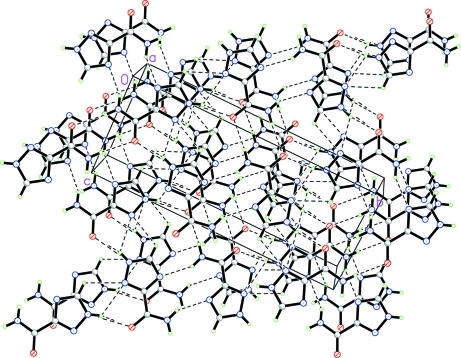
Crystal packing showing the hydrogen bonded interactions as dashed lines.

## supplementary crystallographic information

### Comment

The title compound was obtained by the reaction of methyl 1*H*-1,2,4-triazole-3-carboxylate and ammonium hydroxide. The C=O bond length of 1.2524 (18) Å suggests that the title compound is in the amide form. N—H···O and N—H···N hydrogen bonds link molecules into infinite sheets. These sheets provide the two-dimensional network in and paralell to the *{101}* plane of the cell.

### Experimental

Added 20 ml 25% ammonium hydroxide to methyl 1*H*-1,2,4-triazole-3-carboxylate (20 mmol, 2540 mg) while stirring for 8 h at 353 K. The resulting white precipitate was filtered and washed several times with ethanol and dried in vacuo (yield 85%). Single crystals of C_3_H_4_N_4_O suitable for X-ray diffraction were obtained by slow evaporation of an 50% ethanol solution at room temperature over a period of one week.

### Refinement

The C– and N-bound H atoms were placed in calculated positions and included in the refinement in the riding-model approximation with N—H = 0.86 Å and C—H = 0.93 Å, and with *U*_iso_(H) 1.2*U*_eq_(C,*N*).

### Crystal data

**Table:**

C_3_H_4_N_4_O	*F*_000_ = 232
*M**_r_* = 112.10	*D*_x_ = 1.636 Mg m^−^^3^
Monoclinic, *P*2_1_/*n*	Mo *K*α radiation λ = 0.71073 Å
Hall symbol: -P 2yn	Cell parameters from 1200 reflections
*a* = 3.6944 (4) Å	θ = 2.3–27.5º
*b* = 17.527 (3) Å	µ = 0.13 mm^−^^1^
*c* = 7.0520 (17) Å	*T* = 298 (2) K
β = 94.4670 (10)º	Prism, colourless
*V* = 455.24 (14) Å^3^	0.22 × 0.18 × 0.09 mm
*Z* = 4	

### Data collection

**Table:**

Bruker SMART CCD diffractometer	807 independent reflections
Radiation source: fine-focus sealed tube	657 reflections with *I* > 2σ(*I*)
Monochromator: graphite	*R*_int_ = 0.020
*T* = 298(2) K	θ_max_ = 25.0º
φ and ω scans	θ_min_ = 2.3º
Absorption correction: multi-scan(SADABS; Sheldrick, 1996)	*h* = −4→3
*T*_min_ = 0.972, *T*_max_ = 0.988	*k* = −20→18
2199 measured reflections	*l* = −8→8

### Refinement

**Table:**

Refinement on *F*^2^	Secondary atom site location: difference Fourier map
Least-squares matrix: full	Hydrogen site location: inferred from neighbouring sites
*R*[*F*^2^ > 2σ(*F*^2^)] = 0.035	H-atom parameters constrained
*wR*(*F*^2^) = 0.101	*w* = 1/[σ^2^(*F*_o_^2^) + (0.0653*P*)^2^ + 0.058*P*] where *P* = (*F*_o_^2^ + 2*F*_c_^2^)/3
*S* = 1.05	(Δ/σ)_max_ < 0.001
807 reflections	Δρ_max_ = 0.13 e Å^−^^3^
73 parameters	Δρ_min_ = −0.23 e Å^−^^3^
Primary atom site location: structure-invariant direct methods	Extinction correction: none

### Fractional atomic coordinates and isotropic or equivalent isotropic displacement parameters (Å^2^)

**Table:**

	*x*	*y*	*z*	*U*_iso_*/*U*_eq_	
N1	0.2702 (4)	0.99272 (7)	0.7168 (2)	0.0381 (4)	
H1A	0.3404	1.0240	0.6332	0.046*	
H1B	0.1763	1.0098	0.8163	0.046*	
N2	0.0498 (4)	0.77481 (8)	1.00884 (19)	0.0330 (4)	
H2	0.0162	0.7288	1.0460	0.040*	
N3	0.1784 (4)	0.79443 (7)	0.84073 (18)	0.0329 (4)	
N4	0.0595 (4)	0.89955 (8)	1.01228 (18)	0.0349 (4)	
O1	0.4402 (3)	0.88844 (6)	0.55202 (16)	0.0386 (4)	
C1	0.3067 (4)	0.91813 (8)	0.6923 (2)	0.0289 (4)	
C2	0.1793 (4)	0.87021 (8)	0.8491 (2)	0.0271 (4)	
C3	−0.0168 (5)	0.83716 (9)	1.1079 (2)	0.0360 (4)	
H3	−0.1048	0.8370	1.2279	0.043*	

### Atomic displacement parameters (Å^2^)

**Table:**

	*U*^11^	*U*^22^	*U*^33^	*U*^12^	*U*^13^	*U*^23^
N1	0.0594 (10)	0.0241 (8)	0.0333 (8)	0.0011 (6)	0.0202 (7)	0.0017 (5)
N2	0.0449 (9)	0.0220 (7)	0.0331 (8)	−0.0010 (5)	0.0098 (6)	0.0056 (5)
N3	0.0434 (8)	0.0250 (8)	0.0314 (7)	−0.0006 (5)	0.0093 (6)	0.0010 (5)
N4	0.0494 (9)	0.0259 (8)	0.0310 (8)	0.0004 (6)	0.0145 (6)	0.0004 (5)
O1	0.0566 (8)	0.0267 (6)	0.0347 (7)	0.0030 (5)	0.0176 (5)	−0.0001 (5)
C1	0.0339 (9)	0.0259 (9)	0.0274 (8)	0.0006 (6)	0.0055 (6)	−0.0007 (6)
C2	0.0307 (8)	0.0243 (8)	0.0268 (8)	0.0016 (6)	0.0052 (6)	−0.0009 (6)
C3	0.0495 (10)	0.0292 (9)	0.0308 (8)	−0.0012 (7)	0.0131 (7)	0.0013 (7)

### Geometric parameters (Å, °)

**Table:**

N1—C1	1.3270 (19)	N3—C2	1.3294 (19)
N1—H1A	0.8600	N4—C3	1.326 (2)
N1—H1B	0.8600	N4—C2	1.366 (2)
N2—C3	1.330 (2)	O1—C1	1.2524 (18)
N2—N3	1.3553 (19)	C1—C2	1.493 (2)
N2—H2	0.8600	C3—H3	0.9300
			
C1—N1—H1A	120.0	O1—C1—C2	121.15 (14)
C1—N1—H1B	120.0	N1—C1—C2	114.62 (14)
H1A—N1—H1B	120.0	N3—C2—N4	114.43 (13)
C3—N2—N3	110.03 (13)	N3—C2—C1	121.94 (13)
C3—N2—H2	125.0	N4—C2—C1	123.62 (14)
N3—N2—H2	125.0	N4—C3—N2	110.81 (15)
C2—N3—N2	102.40 (13)	N4—C3—H3	124.6
C3—N4—C2	102.33 (13)	N2—C3—H3	124.6
O1—C1—N1	124.23 (15)		
			
C3—N2—N3—C2	−0.33 (17)	N1—C1—C2—N3	176.10 (15)
N2—N3—C2—N4	0.05 (17)	O1—C1—C2—N4	174.46 (15)
N2—N3—C2—C1	179.30 (13)	N1—C1—C2—N4	−4.7 (2)
C3—N4—C2—N3	0.23 (18)	C2—N4—C3—N2	−0.43 (18)
C3—N4—C2—C1	−179.00 (14)	N3—N2—C3—N4	0.50 (19)
O1—C1—C2—N3	−4.7 (2)		

### Hydrogen-bond geometry (Å, °)

**Table:**

*D*—H···*A*	*D*—H	H···*A*	*D*···*A*	*D*—H···*A*
N1—H1A···O1^i^	0.86	2.21	3.065 (2)	173
N1—H1B···N4^ii^	0.86	2.22	3.010 (2)	154
N2—H2···O1^iii^	0.86	2.07	2.909 (2)	163
N2—H2···N3^iii^	0.86	2.54	3.055 (2)	120

Symmetry codes: (i) −*x*+1, −*y*+2, −*z*+1; (ii) −*x*, −*y*+2, −*z*+2; (iii) *x*−1/2, −*y*+3/2, *z*+1/2.
